# Supplementary material for: Effect of Nut Consumption on Erectile and Sexual Function in Healthy Males: A Secondary Outcome Analysis of the FERTINUTS Randomized Controlled Trial
Source: Nutrients. 2019 Jun 19;11(6):1372. doi: 10.3390/nu11061372 (PMC6627592; doi:10.3390/nu11061372)
Supplement: Supplementary file 1 [file nutrients-11-01372-s001.zip › nutrients-512291-supplementary.docx]

**Table S1.** Average nutrient composition of studied nuts (per 100 g).

| **Nuts (raw)** | **Almonds** | **Hazelnuts** | **Walnuts** |
| --- | --- | --- | --- |
| **NDB Id** | 12061 | 12120 | 12155 |
| **Proximates** |  |  |  |
| **Energy (kcal)** | 597 | 628 | 654 |
| **Total protein (g)** | 21.2 | 15 | 15.2 |
| **Total fat (g)** | 49.9 | 60.8 | 65.2 |
| **Carbohydrate (g)** | 21.6 | 16.7 | 13.7 |
| **Fiber (g)** | 12.5 | 9.7 | 6.7 |
| **Sugars (g)** | 4.4 | 4.3 | 2.6 |
| **Minerals** |  |  |  |
| **Calcium (mg)** | 269 | 114 | 98 |
| **Iron (mg)** | 3.7 | 4.7 | 2.9 |
| **Magnesium (mg)** | 270 | 163 | 158 |
| **Phosphorous (mg)** | 481 | 290 | 346 |
| **Potassium (mg)** | 733 | 680 | 441 |
| **Sodium (mg)** | 1 | 0 | 2 |
| **Zinc (mg)** | 3.1 | 2.5 | 3.1 |
| **Vitamins** |  |  |  |
| **Arginine (g)** | 2.5 | 2.2 | 2.3 |
| **Vitamin C (mg)** | 0 | 6.3 | 1.3 |
| **Thiamin (mg)** | 0.2 | 0.6 | 0.3 |
| **Riboflavin (mg)** | 1.1 | 0.1 | 0.2 |
| **Niacin (mg)** | 3.6 | 1.8 | 1.1 |
| **Vitamin B6 (mg)** | 0.1 | 0.6 | 0.5 |
| **Folate (µg)** | 44 | 113 | 98 |
| **Vitamin B12 (µg)** | 0 | 0 | 0 |
| **Vitamin A (RAE) (µg)** | 0 | 1 | 1 |
| **α-tocopherol (mg)** | 25.6 | 15 | 0.7 |
| **Vitamin D (D2+D3) (µg)** | 0 | 0 | 0 |
| **Vitamin K (µg)** | 0 | 14.2 | 2.7 |
| **Lipids** |  |  |  |
| **SFA (g)** | 3.8 | 4.5 | 6.1 |
| **MUFA (g)** | 31.6 | 45.7 | 8.9 |
| **PUFA (g)** | 12.3 | 7.9 | 47.2 |
| **TFA (g)** | <0.1 | ND | ND |
| **Cholesterol (mg)** | 0 | 0 | 0 |

Abbreviations: SFA: saturated fatty acid, MUFA: monounsaturated fatty acid, PUFA: polyunsaturated fatty acid, ND: no data reported, RAE: retinol activity equivalents, TFA: trans fatty acid. Data are from US Department of Agriculture Nutrient Database. Available from: <https://ndb.nal.usda.gov/ndb/search/list> [33]. Last accessed on 25^th^ April 2019.
